# Registered nurses: can our supply meet the demand during a disaster?

**DOI:** 10.1186/s12912-021-00794-x

**Published:** 2022-01-04

**Authors:** Yin Li, Jason M. Hockenberry, Jiaoan Chen, Jeannie P. Cimiotti

**Affiliations:** 1 Nell Hodgson Woodruff School of Nursing, Emory University, 1520 Clifton Road NE, Atlanta, Georgia 30322-4027 USA; 2 Department of Health Policy and Management, School of Public Health, Yale University, 60 College Street, New Haven, CT 06520-0834 USA; 3 Shenzhen BGI Co, Ltd, Shenzhen, China

**Keywords:** Natural disaster, Registered nurses, Supply, Demand

## Abstract

**Background:**

Death and destructions are often reported during natural disasters; yet little is known about how hospitals operate during disasters and if there are sufficient resources available for hospitals to provide ongoing care during these catastrophic events. The purpose of this study was to determine if the State of New Jersey had a supply of registered nurses (RNs) that was sufficient to meet the needs of hospitalized patients during a natural disaster – Hurricane Sandy.

**Methods:**

Secondary data were used to forecast the demand and supply of New Jersey RNs during Hurricane Sandy. Data sources from November 2011 and 2012 included the State Inpatient Databases (SID), American Hospital Association (AHA) Annual Survey on hospital characteristics and staffing data from New Jersey Department of Health. Three models were used to estimate the RN shortage for each hospital, which was the difference between the demand and supply of RN full-time equivalents.

**Results:**

Data were available on 66 New Jersey hospitals, more than half of which experienced a shortage of RNs during Hurricane Sandy. For hospitals with a RN shortage in ICUs, a 20% increase in observed RN supply was needed to meet the demand; and a 10% increase in observed RN supply was necessary to meet the demand for hospitals with a RN shortage in non-ICUs.

**Conclusion:**

Findings from this study suggest that many hospitals in New Jersey had a shortage of RNs during Hurricane Sandy. Efforts are needed to improve the availability of nurse resources during a natural disaster.

## Background

In recent years, the U.S. has seen an increase in the number of life-threatening hurricanes, which is most likely due to global climate change [1]. These storms significantly impact the functionality of hospitals and health care systems in areas of storm surge. One notable storm was Hurricane Sandy, a storm that ravaged the entire eastern seaboard of the U.S. with deadly and destructive storm activity that lasted from October 22 to October 31, 2012. Hurricane Sandy caused significant damage in 24 U.S. states and the District of Columbia and it completely disrupted the overall function of hospitals and health care systems especially in the states of New Jersey and New York [2–4]. In New York City alone it was reported that approximately 6300 patients from 37 health care facilities were evacuated, with more than $2 billion in damage-related costs [4].

During natural disasters, such as hurricanes, registered nurses (RNs) are the key responders and the providers of quality care [5]. A sustainable RN workforce play a critical role in maintaining the surge capacity of a health care system and in meeting the increased needs for patient care, such as triaging patients, providing treatment, and administering emergency care [6]. Without a robust RN workforce during a disaster, the provision of safe, sustainable, and effective care is impossible [7–9].

While occurring at an increasing rate, disasters are a rare and time concentrated event in the U.S. As such little is known about the availability of RNs and hospital-based RNs staff during a disaster. Further, we know little about hospitals and their ability to identify sources of additional RN staff that might be required to meet the increased demand for care during a disaster [10]. To fill this knowledge gap, we examined the supply of RNs in the hospitals of New Jersey during Hurricane Sandy to determine if the RN workforce was able to meet the increased demand for health care services. The results from this study provide substantial evidence to inform workforce planning during a disaster.

## Literature review

There is substantial scientific evidence to suggest that lower RN staffing levels are associated with poor patient outcomes, such as higher rates of patient mortality and healthcare-associated infection [11–15]; an association that becomes even more complex when there is an increased demand for nursing care such as during a disaster [6]. Although few studies have examined the impact of nurse staffing on patient outcomes during a disaster, there is some research to suggest that during a mass casualty incident a shortage of nurses will result in longer lengths of stay and higher inpatient charges [16].

Hospital leadership and public health officials often seek RNs to help in surge areas during a disaster. Recently, the Texas Board of Nursing issued more than 600 temporary licenses to nurses from other states to support disaster relief efforts after Hurricane Harvey [17]; and Florida Governor Scott tweeted that, “Florida needs 1,000 nurses volunteer nurses to help …” during Hurricane Irma. However, the mobility of RNs is often limited due to jurisdictional licensure restrictions, which prevents RNs from providing care in a timely manner during a disaster [6]. Although many states accept RNs from other states to support surge areas during a “declared” disaster, RNs may not be able to work immediately due to the administrative process of license verification or acquiring a temporary license [3]. These processes hinder the delivery of timely, high-quality care.

Estimating the need for RNs during disaster is an important yet unexplored aspect of the RN workforce that is necessary to inform policy change and improve RN mobility. In an effort to inform stakeholders, we examined several models of RN staffing in New Jersey hospitals during Hurricane Sandy to determine if there was a shortage of RNs, and if so, how many RNs would have been needed to address that shortage.

## Method

### Design and data

This study was a cross-sectional analysis using RN staffing data, patient data, and hospital data from 2011 and 2012 that forecasted the supply and demand of nurses during Hurricane Sandy. The RN staffing data for each New Jersey hospital were obtained from the New Jersey Department of Health (NJDOH). The NJDOH collects daily ratio of patients per RNs from all hospitals by unit type, which are further aggregated, averaged, and reported at the quarter level. These quarterly patient to RN ratios were used for this analysis.

Patient data were obtained from the State Inpatient Databases (SID). The SID was developed for the Healthcare Costs and Utilization Project and sponsored by the Agency for Healthcare Research and Quality. Although this dataset includes numerous data on patients’ demographics, diagnoses, procedures, and so on, only the number of inpatient discharges from New Jersey hospitals were used in this study.

Hospital data were obtained from the American Hospital Association (AHA) Annual Survey. As a census survey of the U.S. hospitals, the AHA Annual Survey collects comprehensive characteristics of hospitals, including but not limited to staffing, ownership, beds, geographic characteristics, and accreditations and so on.

### Sample

Four out of the 72 acute care hospitals in New Jersey without data on RN staffing were excluded. Another three hospitals were reported as one system. Thus, 66 New Jersey hospitals were included for further analysis. Data on all adult and pediatric patients who were discharged from one of the study hospitals during November 2011 and November 2012 were included in the analyses.

### Variables and measurements

#### Hospital characteristics

The hospital characteristics obtained from the 2012 AHA Annual Survey included bed size, high-technology status, teaching status, safety-net hospital, and Magnet designated status. The bed size was a categorical variable, with the categories of <=100 beds, 101–250 beds, and > 250 beds. The high-technology status was a binary variable (yes or no) and defined as whether a hospital implemented electronic health records or not. Teaching status was also a binary variable (yes or no) and was defined as if a hospital was approved to participate in residency and/or internship training by the Accreditation Council for Graduate Medical Education, had a medical school affiliation through the American Medical Association, was a member of the Council of Teaching Hospitals of the Association of American Medical Colleges, had an internship approved by American Osteopathic Association, or had an residency approved by American Osteopathic Association. The safety-net status was a binary variable (yes or no), and a hospital was defined as a safety-net hospital if it provides uncompensated care for the uninsured and most vulnerable patients [18]. The Magnet designated status was a binary variable (yes or no); a hospital is accredited as a Magnet hospital for good nursing care based on a list obtained from the American Nurses Credentialing Center.

#### Staffing

The average patient to RN ratio of the fourth quarter (October, November, and December) in 2011 and 2012 were used for this analysis. These data were converted to estimate the RN full-time equivalents (FTEs) in November for 2011 and 2012. All hospital units that provide adult and pediatric acute care were categorized into two types: intensive care unit (ICU) and non-ICU. The ICUs included adult ICU, neonatal ICU, and pediatric ICU; and non-ICU included adult open psychiatric unit, adult closed psychiatric, child/adolescent closed psychiatric, intermediate, medical surgical, neonatal-intermediate, normal newborn nursery, obstetrics, pediatrics, and substance abuse.

#### Patient characteristics

Data on patient characteristics were derived from the SID and included admission unit, length of stay, and a discharge date that occurred in November 2011 or November 2012. Patients were categorized into two groups based on unit type -- ICU and non-ICU. The length of stay and the number of discharges were aggregated to the hospital level where the average length of stay and the total number of discharges for each hospital were computed and used in the analyses.

#### Storm severity of hurricane Sandy

The counties of New Jersey were categorized as moderate, high, and very high impacted areas based on the number of people exposed to surge, the dollars in wind damage, and the volume of rain [19] (Table 1).

**Table 1:** A list of counties based on the storm severity of hurricane sandy

Storm Severity	County
**Very High Areas:** > 10,000 people exposed to surge	Atlantic, Ocean, Monmouth, Middlesex, Union, Essex, Bergen, Hudson, Cape May
**High Areas:** 5000–10,000 people exposed to surge; or > $100 million in wind damage; or > 8″ of rain	Cumberland, Salem, Gloucester, Camden, Burlington, Mercer, Somerset, Morris, Passaic
**Moderate Areas:** 100–500 people exposed to surge; or $10–100 million in wind damage;or 4–8″ of rain	Sussex, Warren, Hunterdon,

### Analysis

Data from the SID, AHA, and NJDOH were merged to address the purpose of the study. Our models of forecasting were based on previous work that examined the demand for nurses [20]. Specifically, three models were developed to estimate if the supply of nurses in New Jersey hospitals was able to the meet the demand during Hurricane Sandy.

#### Model 1

We first calculated the observed RN FTEs in November 2012 using the following formula [21]:

[1] *Observed RN FTEs Nov. 2012=* $\frac{\frac{1}{the\ number\ of\ patient s\  per\  RN\  in\ Quarter\ 4\  of\ 2012}\ast the\ total\ patient\ days\ in\  Nov.2012\ast 24}{40\ast 4\ast 0.85}$

Where *the number of patients per RN in the 4th quarter of 2012*; *the total patient days in November 2012* were calculated by multiplying the average length of stay by the total number of discharges in each hospital in November 2012. The denominator of this formula provided the total observed nursing hours in November 2012. By dividing these nursing hours by 40 h per week, 4 weeks per month, and an assumption of 0.85 productive hours, we obtained the *observed RN FTEs* for each hospital in November 2012.

In an effort to determine if the observed number of RN FTEs were able to meet the demand for nurses, we first estimated the *expected RN FTEs* in November 2012 by using the observed RN FTEs of November 2011 as baseline using the following formula:

[2] *Expected RN FTEs in Nov.2012=* $\frac{Observed\  RN\  FTEs\ in\  Nov.2011}{Total\ patient\ days\ in\  Nov.2011}$ * Total patient days in Nov. 2012

Where the *observed RN FTEs in November 2011* were calculated using formula [1]; the *total patient days in November 2011* were calculated by multiplying the average length of stay by the total number of discharges in each hospital in November 2011. The results of dividing the *observed RN FTEs in November 2011* by the *total patient days in November 2011* provided the observed RN FTEs per patient day in November 2011. Assuming this rate was not changed in November 2012, the expected RN FTEs in November 2012 were estimated by multiplying the rate by the *total patient days in November 2012*.

To determine whether a hospital had a shortage of RN FTEs, the *expected RN FTEs in November 2012* were subtracted from the *Observed RN FTEs in November 2012*:

*Difference 1 = Observed RN FTEs in November 2012 - Expected RN FTEs in November 2012*

A negative difference indicating that the observed RN FTEs was smaller than the expected RN FTEs in November 2012, suggesting of a shortage of nurses.

#### Model 2

Using November 2012 data on the hospitals with a shortage of RN FTEs, we estimated how an increase in the observed RN FTEs could meet hospital demand. This was calculated through a 10% increase of the observed RN FTEs in November 2012:

Difference 2 = Observed RN FTEs in November 2012*110% - Expected RN FTEs in November 2012

#### Model 3

Similar to Model 2, using November 2012 data from hospitals with a shortage of RN FTEs, we estimated how an increase in the observed RN FTEs could meet hospital demand. This model was calculated through a 20% increase in the observed RN FTEs in November 2012:

Difference 3 = Observed RN FTEs in November 2012*120% - Expected RN FTEs in November 2012

These three model estimations were conducted separately by unit type (ICU and non-ICU) to estimate the shortage of RN FTEs. Descriptive analyses were used to summarize the shortage of RN FTEs by county. All analyses were conducted using STATA/MP 15.1 (StataCorp, College Station, TX).

## Results

### Hospital characteristics

There were 39 (59.1%) out of 66 hospitals were located in very high impact areas, 23 (34.8%) in high impact areas, and 4 (6.1%) in moderate impact areas (Table 2). More than half (*n* = 40, 60.6%) of the total hospitals were relatively large with more than 250 beds. There were 40 (60.6%) teaching hospitals, 54 (81.8%) partially or fully implemented electronic health record system, 28 (27.3%) safety-net hospitals, and 26 (39.4%) Magnet hospitals.

**Table 2:** Characteristics of New Jersey Hospitals Included in the Study (n = 66)

Characteristics	Total (***n*** = 66)	Very High Impact (***n*** = 39)	High Impact (***n*** = 23)	Moderate Impact (***n*** = 4)
**Bed size**				
< 100	2 (3.0%)	0 (0.0%)	2 (8.7%)	0 (0.0%)
101–250	24 (36.4%)	13 (33.3%)	7 (30.4%)	4 (100.0%)
> =251	40 (60.6%)	26 (66.7%)	14 (60.9%)	0 (0.0%)
**Teaching status**				
No	26 (39.4%)	15 (38.5%)	9 (39.1%)	2 (50.0%)
Yes	40 (60.6%)	24 (61.5%)	14 (60.9%)	2 (50.0%)
**High-tech hospital**				
No	12 (18.2%)	6 (15.4%)	5 (21.7%)	1 (25.0%)
Yes	54 (81.8%)	33 (84.6%)	18 (78.3%)	3 (75.0%))
**Safety-net hospital**				
No	48 (72.7%)	28 (71.8%)	16 (69.6%)	4 (100.0%)
Yes	18 (27.3%)	11 (28.2%)	7 (30.4%)	0 (0.0%)
**Magnet hospital**				
No	40 (60.6%)	22 (56.4%)	15 (65.2%)	3 (75.0%)
Yes	26 (39.4%)	17 (43.6%)	8 (34.8%)	1 (25.0%)

Data used for developing this table are from the 2012 AHA annual survey

### Nurse shortages in ICUs

Twenty-nine of the 66 (46.9%) New Jersey hospitals in the very high and high impacted areas had a negative difference between the observed and expected RN FTEs during November 2012 in ICUs, indicating a shortage of RN FTEs (Table 3).

**Table 3:** Estimates of RN FTEs Shortage in ICUs by County (*N* = 29)

	Shortage of RN FTEs		
	Model 1^**a**^	Model 2^**b**^	Model 3^**c**^
**Very High Impacted Areas (*n*** **= 18)**			
Atlantic	-20.2	-3.0	14.2
Bergen	-33.4	2.3	38.0
Essex	-45.5	-19.6	6.3
Hudson	-52.6	-44.2	-35.9
Middlesex	-13.4	10.6	34.6
Monmouth	-39.0	- 19.5	0
Ocean	-16.8	- 1.4	14.0
Union	-18.0	7.8	33.5
**High Impacted Areas (*n*** **= 11)**			
Camden	-35.3	25.1	85.5
Cumberland	-11.2	11.2	33.6
Mercer	-75.3	- 53.1	-30.9
Morris	-11.5	0.9	81.6
Passaic	-11.3	0.5	13.0
Salem	-8.8	2.8	3.2

*a = observed RN FTEs; b = 10% increase in observed RN FTEs; c = 20% increase in observed RN FTEs*

#### Model 1

Among the 29 hospitals with a shortage, 18 (62%) were in the very high impact storm area and 11 (38%) were in the high impact area. For the 18 hospitals in the very high impact area, the shortage of RN FTEs ranged from − 13.4 to − 52.6. The 11 hospitals in the high impact storm area had a shortage of RN FTEs that ranged from −- 8.8 to − 75.3.

#### Model 2

In this model we increased the observed number of RN FTE by 10% and still found that there were nurse shortages. Hospitals in eight counties continued to have an estimated shortage of RN FTEs despite the 10% increase in RN FTEs. The hospitals that continued to have a shortage of RN FTEs were geographically located in five very high impact counties (Atlantic, Essex, Hudson, Monmouth, and Ocean) and one high impact county (Mercer).

#### Model 3

In this model we increased the observed number of RN FTEs by 20% and found that all hospitals had an adequate number of RN FTEs except for Hudson County in the very high impact storm area and Mercer county in the high impact storm area. It should be noted that Hudson County lies along the lower Hudson River and has been designated as a flood zone. During Hurricane Sandy Hudson County was flooded due to the storm surge through New York Bay and subsequently into the Hudson River.

### Nurse shortages in non-ICUs

Similar to the ICUs, 37 out of the 66 (60.6%) New Jersey hospitals in the very high and high impacted areas had a negative difference between the observed and expected RN FTEs during November 2012 in non-ICUs, indicating a shortage of RN FTEs (Table 4).

**Table 4:** Estimates of RN FTEs in non-ICUs by County (*N* = 37)

	Shortage of RN FTEs		
	Model 1^**a**^	Model 2^**b**^	Model 3^**c**^
**Very High Impact (*n*** **= 26)**			
Atlantic	-16.0	3.2	22.4
Bergen	-32.4	8.3	49.1
Essex	-6.3	9.4	25.0
Hudson	-1.1	9.2	19.6
Middlesex	-6.5	12.2	30.9
Monmouth	-26.8	-8.1	10.5
Ocean	-3.7	5.5	14.6
Union	-9.6	3.1	15.8
**High Impact (*n*** **= 11)**			
Burlington	-8.2	1.1	10.4
Cumberland	-30.0	-5.3	19.5
Mercer	-22.4	-6.9	8.7
Morris	-3.2	27.9	59.1
Passaic	-0.4	3.7	7.7
Salem	-4.5	-0.8	2.9
Somerset	-10.7	6.8	24.2

*a = observed RN FTEs; b = 10% increase in observed RN FTEs; c = 20% increase in observed RN FTEs*

#### Model 1

Among the 37 hospitals with a shortage, 26 (70.3%) hospitals were in the very high impact storm area and 11 (29.7%) were in the high impact area. Hospital-based non-ICUs had less of an RN FTE shortage when compared to the shortage of RN FTEs in hospital-based ICUs. For the hospitals in the very high impact area, the shortage of RN FTEs ranged from − 1.1 to − 32.4. The hospitals in the high impact area had a shortage of RN FTEs that ranged from − 0.4 to − 30.0.

#### Model 2

In this model we increased the observed number of RN FTEs by 10% and still found that there were nurse shortages. Hospitals in three counties continued to have an estimated shortage of RN FTEs despite the 10% increase in RN FTEs. These hospitals that continued to have a shortage of RN FTEs were geographically located in a very high impact county (Monmouth) and high impact counties (Cumberland, Mercer, and Salem).

#### Model 3

In this model we increased the observed number of RN FTEs by 20% and found that all hospitals had an adequate number of RN FTEs.

## Discussion

The findings from this study suggest that many of New Jersey hospitals had experienced a shortage of nurses during Hurricane Sandy. This occurred statewide, regardless of the storm severity, where there were reports that hospitals were operating at full capacity with power outages that lasted for days [22]. Hospitals in the very high and high impact storm areas might have experienced the reported shortage of nurses due to the surge, while the hospitals in the moderate impact areas might have experienced a shortage due to the possibility that patients were transferred in from other hospitals, especially from those hospitals in very high and high impact areas. All of this is evidence that the ongoing collection of nurse workforce data is essential if we are to be prepared for unforeseen disasters.

During the time of Hurricane Sandy, the hospitals of New Jersey did not allow nurses with an out-of-state license to practice in New Jersey during a disaster or emergency. Thus, the hospitals and health care systems in these areas relied on their own resources to maintain the workforce at surge capacity during a natural disaster. Although hospitals might have recruited agency nurses or travel nurses to staff their units during Hurricane Sandy, the findings of this study suggest a widespread shortage of nurses in the hospitals statewide. Moreover, nurses in these areas reported a high level of stress from being assigned to an overwhelming number of patients that they did not feel they were able to manage safely [3].

Previous studies have shown that a licensure compact was associated with an increase of worker’s inter-state mobility and supply. These studies included a variety of health care professionals such as dentists, physicians, and surgeons, but few studies have examined relevant issues among nurses [23–27]. In order to improve the mobility of nurses especially during disaster, the Nurse Licensure Compact was introduced by the National Council of State Boards of Nursing in 1999. The Nurse Licensure Compact allows registered nurses and licensed practical nurses with a valid license from a compact-state to practice in another compact-state without acquiring an additional license from that state [28]. By allowing nurses to practice across state lines, the compact can help increase the number of available nurses to support surge areas immediately during a natural or man-made disaster. Unfortunately, New Jersey was not a compact state during Hurricane Sandy and limitations were placed on nurses with an out-of-state license, which could limit nurses’ ability to cross state lines into New Jersey to practice. However, whether or not the compact can maintain the surge capacity of a health care system during a disaster has gone largely unexplored and warrants further investigation.

This study had a few limitations. First, nurse staffing data were obtained from the New Jersey Department of Health; data that are reported quarterly. More granular data such as monthly, weekly, or even daily nurse staffing would have allowed for better estimates of RN shortages. Second, we limited our research to one state (New Jersey) during one storm (Hurricane Sandy) and as such our findings might not be generalizable to hospitals in other states and during other disasters. Third, our models are based on the assumption that the quarterly staffing data retrieved from the New Jersey Department of Health included all possible sources of nurses, such as nurses provided through agencies. Despite these limitations, this study provides important evidence on nurse staffing during a natural disaster; evidence that can inform future workforce planning and policies that aim at meeting the surge capacity of hospitals and health care systems and improving patient outcomes during a natural or man-made disaster.

## Conclusion

This study estimated that more than half of the hospitals in the state of New Jersey had a shortage of RNs during Hurricane Sandy. Natural disasters are unexpected events that can result in widespread disruption in the delivery of health care services. States need to be proactive in an effort to sustain health care delivery during these disasters and to have nurses available to provide a seamless delivery of care. We have recently witnessed this with the ongoing COVID-19 pandemic where states nationwide are struggling to maintain an adequate supply of nurses. Ensuring an adequate and flexible nurse workforce is essential during a disaster. The nurse licensure compact is a model that should be adopted by states nationwide, which can ensure the ability of a workforce to practice across state lines in the time of greatest need. Thus, when the next disaster strikes, we can be confident that there will be a sufficient supply of nurses on the frontlines of care.

## Data Availability

The datasets analyzed during the current study are available in the following: State Inpatient Databases: https://www.hcup-us.ahrq.gov/sidoverview.jsp American Hospital Association Annual Survey: https://www.ahadata.com/aha-annual-survey-database New Jersey Department of Health: https://healthapps.state.nj.us/nursestaffing/quarterly.aspx

## Abbreviations

RN: Registered Nurses
NJDOH: New Jersey Department of Health
SID: State Inpatient Databases
AHA: American Hospital Association
FTEs: Full-time equivalents
ICU: Intensive Care Units
