# Correction: Registered nurses: can our supply meet the demand during a disaster?

**DOI:** 10.1186/s12912-022-01079-7

**Published:** 2022-11-10

**Authors:** Yin Li, Jason M. Hockenberry, Jiaoan Chen, Jeannie P. Cimiotti

**Affiliations:** 1 Nell Hodgson Woodruff School of Nursing, Emory University, 1520 Clifton Road NE, Atlanta, Georgia 30322-4027 USA; 2 Department of Health Policy and Management, School of Public Health, Yale University, 60 College Street, New Haven, CT 06520-0834 USA; 3 Shenzhen BGI Co, Ltd, Shenzhen, China

**Correction: BMC Nurs 21, 7 (2022)**

**https://doi.org/10.1186/s12912-021-00794-x**

Following publication of the original article [1], the authors flagged an issue with one of the provisions of the dataset they’ve used. The article is an analysis of the State Inpatient Database (SID), a restricted-access, publicly available dataset that is maintained by the Agency for Healthcare Research and Quality (AHRQ). One of the provisions of the SID Data Use Agreement (DUA), is that no individual establishments may be identified directly or by inference. This measure was put in place by AHRQ to protect institutions’ and individual patients’ privacy and to prevent the potential disclosure of personal information.

In the original Tables 3 and 4 and Figures 1 and 2, there is some data which is in violation of this provision. The authors apologize for the oversight and the inadvertent failure to comply with the Data Use Agreement. While the authors are fully confident that nothing in the original article could be used to identify individuals or their personal information, necessary action was taken in order to comply with the AHRQ guidelines:

- Tables 3 and 4 were revised.
- Figures 1 and 2 were removed.
- In Table 2, a footnote was added with the statement “Data used for developing this table are from the 2012 AHA annual survey”.
- The subsections ‘Nurse shortages in ICUs’ and ‘Nurse shortages in non-ICUs’ in the ‘Results’ were revised.

**Table 3:** Estimates of RN FTEs Shortage in ICUs by County (*N*=29)

	Shortage of RN FTEs		
	Model 1^a^	Model 2^b^	Model 3^c^
**Very High Impacted Areas (*n*=18)**			
Atlantic	-20.2	-3.0	14.2
Bergen	-33.4	2.3	38.0
Essex	-45.5	-19.6	6.3
Hudson	-52.6	-44.2	-35.9
Middlesex	-13.4	10.6	34.6
Monmouth	-39.0	-19.5	0
Ocean	-16.8	-1.4	14.0
Union	-18.0	7.8	33.5
**High Impacted Areas (*n*=11)**			
Camden	-35.3	25.1	85.5
Cumberland	-11.2	11.2	33.6
Mercer	-75.3	-53.1	-30.9
Morris	-11.5	0.9	81.6
Passaic	-11.3	0.5	13.0
Salem	-8.8	2.8	3.2

^a^ observed RN FTEs, ^b^ 10% increase in observed RN FTEs, ^c^ 20% increase in observed RN FTEs

**Table 4:** Estimates of RN FTEs in non-ICUs by County (*N*=37)

	Shortage of RN FTEs		
	Model 1^a^	Model 2^b^	Model 3^c^
**Very High Impact (*n*=26)**			
Atlantic	-16.0	3.2	22.4
Bergen	-32.4	8.3	49.1
Essex	-6.3	9.4	25.0
Hudson	-1.1	9.2	19.6
Middlesex	-6.5	12.2	30.9
Monmouth	-26.8	-8.1	10.5
Ocean	-3.7	5.5	14.6
Union	-9.6	3.1	15.8
**High Impact (*n*=11)**			
Burlington	-8.2	1.1	10.4
Cumberland	-30.0	-5.3	19.5
Mercer	-22.4	-6.9	8.7
Morris	-3.2	27.9	59.1
Passaic	-0.4	3.7	7.7
Salem	-4.5	-0.8	2.9
Somerset	-10.7	6.8	24.2

^a^ observed RN FTEs, ^b^ 10% increase in observed RN FTEs, ^c^ 20% increase in observed RN FTEs

The revised Tables 3 and 4 are given below and the original article has been corrected.
